# Flow transport and not ejection fraction determines blood stasis in patients with impaired left ventricular systolic function

**DOI:** 10.14814/phy2.70351

**Published:** 2025-07-04

**Authors:** Pablo Martinez‐Legazpi, Javier Bermejo, Juan C. del Alamo

**Affiliations:** ^1^ Department of Mathematical Physics and Fluids, Facultad de Ciencias Universidad Nacional de Educación a Distancia, UNED and CIBERCV Madrid Spain; ^2^ Department of Cardiology Hospital General Universitario Gregorio Marañón; Facultad de Medicina, Universidad Complutense de Madrid, Instituto de Investigación Sanitaria Gregorio Marañón and CIBERCV Madrid Spain; ^3^ Mechanical Engineering Department, Division of Cardiology, and Center for Cardiovascular Biology University of Washington Seattle Washington USA

**Keywords:** blood stasis, doppler‐echocardiography, intraventricular flow, left ventricle, thrombosis

## Abstract

Impaired left ventricular (LV) systolic function is a risk factor for intraventricular thrombosis and cardioembolism. However, below a given threshold, LV ejection fraction (EF) poorly predicts these events, suggesting the existence of additional sources of variability. We introduce queue models of LV blood transit connecting flow component analysis and residence time (RT) mapping. These models yield closed‐form expressions for the average RT of blood in the LV as a function of (1) EF, (2) direct flow (DF), and (3) residual volume (RV). Models' performance was tested against RT obtained from vector flow mapping in 332 subjects, including controls and patients with acute myocardial infarction (AMI), hypertrophic (HCM), and dilated cardiomyopathy (DCM). Queue models show RT is increasingly sensitive to DF as EF decreases, contradicting the traditional view of large DF as a teleological advantage. Instead, RT is minimized when blood transits in a first‐in‐first‐out (FIFO) manner, while DF short‐circuits the FIFO pattern, prolonging RT for other flow components. FIFO models showed a good performance in assessing RT in the studied subjects. Our results show that large DFs increase blood stasis when EF is low. These models also explain why EF is a poor marker of the risk of intraventricular thrombosis.

## 1 INTRODUCTION

Increased blood stasis is a well‐known risk factor for left ventricular (LV) mural thrombosis. When combined with endocardial damage and systemic procoagulant states (e.g., chronic heart failure (HF) or acute myocardial infarction (AMI)), increased LV stasis places patients at particular risk for intraventricular thrombosis and cardioembolism. In the absence of atrial fibrillation, thromboembolic events in the setting of AMI are usually due to LV thrombus formation (Delewi et al., 2012). Also, patients with nonischemic dilated cardiomyopathy (NIDCM) have a 3–8 times higher risk of ischemic stroke than age‐matched individuals (Lip et al., 2012). In these settings, a low LV ejection fraction (EF) is thought to increase the risk of LV mural thrombosis because impaired global systolic chamber function may exacerbate intracavitary stasis (Pullicino et al., 2006). Consequently, EF inversely correlates with the incidence of stroke in HF patients and AMI survivors (Dries et al., 1997). However, this correlation excludes large demographic groups, and the clinical trials of anticoagulant agents on HF patients with reduced EF have yielded contradictory results (Zannad et al., 2018). Recent pilot prospective studies have also shown that, below a certain threshold, EF does not account for the risk of cardioembolism, suggesting that flow transport efficiency influences LV blood stasis in addition to global systolic function (Bermejo et al., 2015; Rodriguez‐Gonzalez et al., 2024, 2025).

Flow in the LV follows complex swirling patterns connecting the inflow and the outflow jets. The discovery of these patterns prompted numerous investigations focusing on their origin and teleology (Kilner et al., 2000). LV flow patterns define the pathlines of blood particles, partitioning the chamber into four regions with distinct transport dynamics: direct flow (DF), retained inflow (RI), delayed ejection (DE), and residual volume (RV) (Eriksson et al., 2010). These regions are heavily altered in cardiomyopathies, leading to large residual volumes (Svalbring et al., 2016). Due to its effect on transport efficiency, DF—the volume that enters and exits the chamber within the same cardiac cycle—is traditionally interpreted as teleologically beneficial (Svalbring et al., 2016). This interpretation has been favored by observation in patients with NIDCM, where increased LV chamber sphericity and contractility changes coincide with larger swirling flow trajectories, reduced DF, and increased RV (Eriksson et al., 2013). Analysis of flow transport in the setting of an AMI yields similar results (Demirkiran et al., 2022; Stoll et al., 2019).

Residence time (RT) is defined as the average time spent by blood particles inside a chamber. Methods have been developed for mapping RT from vector flow data obtained either from echocardiography or the phase contrast sequences of cardiac magnetic resonance (Rossini et al., 2016). Although mean RT of blood in the LV and EF are inversely related (Costello et al., 2018), this association is imperfect, and high RT shows far better accuracy for predicting mural thrombosis, silent brain infarcts, and cardioembolic strokes in patients with NIDCM and AMI than low EF (Rodriguez‐Gonzalez et al., 2024, 2025). RT is intrinsically related to the compartmentalization of blood transit through the LV chamber (Hendabadi et al., 2013). Intuitively, increased RV and reduced DF components are associated with a high RT, but there is no mathematical representation of such correspondence (Eriksson et al., 2013).

The present study was designed to assess the interplay between blood stasis, flow transit components, and global systolic chamber function of the LV. For this purpose, we developed simple queue models of transit. Based on these models, this relationship is less intuitive than previously thought.

## 2 METHODS

### 2.1 Study population

We selected 332 subjects from previous studies (Benito et al., 2019; Rodriguez‐Gonzalez et al., 2024, 2025), including 90 patients with nonischemic dilated cardiomyopathy (DCM), 93 patients with hypertrophic cardiomyopathy (HCM), 82 patients with acute myocardial infarction (AMI), and 67 healthy volunteers (Controls, with absence of known or suspected cardiovascular disease and normal electrocardiographic and ultrasound examinations).

### 2.2 Blood transport and stasis mapping from ultrasound imaging

Echocardiograms were performed using Vivid 7/E95 scanners and broadband transducers (GE Healthcare) and the two‐dimensional (2D + t) blood flow field in the LV was obtained using vector flow mapping (VFM), as described elsewhere (Garcia et al., 2010). Inputs for the VFM are a color‐Doppler acquisition (~10 beats) followed by a 2D cine‐loop (~5 beats) at high frame rate without moving the ultrasound probe. By integrating the fluid mechanics continuity equation, imposing no‐penetration condition at the chamber walls, VFM yields the crossbeam flow velocity. The 2D + t blood flow fields from the VFM, $\vec{v\;}$, were used to integrate a forced advection equation to obtain the RT maps of blood inside the LV (Rossini et al., 2016):

(1) $$\frac{\partial \mathrm{RT}}{\partial t}+\nabla \cdot \left(\vec{v}\mathrm{RT}\right)=1$$

We integrated equation 1 during 8 cycles, the estimate to washout the entire blood pool in a healthy LV (Benito et al., 2019), and collected the averaged value of RT at the end of last cycle (Benito et al., 2019; Rodriguez‐Gonzalez et al., 2024, 2025). Equation 1, without unit forcing, was also integrated both forward and backwards in time during two cycles, to track the advection of a scalar, ϕ, allowing us to determine size and location of blood transport regions (DF, RI, DE, and RV) at end‐diastole (ED) (Postigo et al., 2022).

### 2.3 Queue models for LV RT

We derived several models to estimate the mean RT in the LV, accounting for different LV transit patterns and microscopic mixing, using EF and DF as independent variables. The full derivation of these models can be found in the Appendix S1. We summarize these models in order of increasing complexity:

#### 2.3.1 Perfect mixing transit model

**FIGURE 1:**
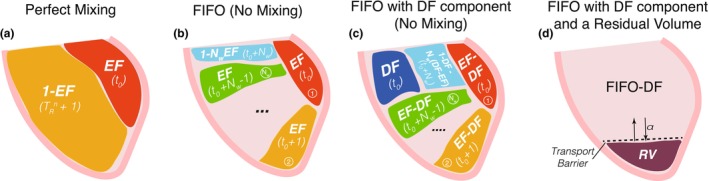
Sketch of the queue models. From left to right: (a) Perfect Mixing, (b) First‐in‐First Out (FIFO), (c) FIFO with Direct Flow (FIFO‐DF) and (d) FIFO with DF and a residual volume (RV). The size of each region, scaled to the end‐diastolic volume, is displayed in capital letters. The RT for each region is shown in lowercase. $N_{w}$, washout number; $t_{0}$, diastole duration; EF, ejection fraction; α, fraction of blood within the RV that is exchanged with the rest of the LV.

This model assumes complete mixing of blood in the LV at the end of diastole, leading to an equation for mean RT based solely on the value of EF, ${\mathrm{RT}}_{\mathrm{LV},\text{mixed}}=t_{0}+\frac{1-\mathrm{EF}}{\mathrm{EF}}$. This equation provides a theoretical baseline where RT approaches $t_{0}$, a model parameter that measures the end‐diastolic RT of the blood that enters the LV each cycle, when EF approaches 1 (Figure 1a).

#### 2.3.2 Zero‐mixing FIFO model

For any given EF value, RT is minimized when blood transits the chamber following a first‐in first‐out (FIFO) flow pattern, where blood with the longest RT immediately exits the chamber. To find RT under a FIFO pattern, we divided the LV into $N_{w}=\mathrm{int}\left(\frac{1}{\mathrm{EF}}\right)$ equal queued compartments of volume $V_{i,\mathrm{EF}}=\mathrm{EF}\cdot \mathrm{EDV}$. The resulting RT depends on EF and $N_{w}$ as ${\mathrm{RT}}_{\mathrm{LV},\text{FIFO}}=N_{w}+t_{0}-\frac{\mathrm{EF}\left(N_{w}+1\right)N_{w}}{2}$, (Figure 1b).

#### 2.3.3 FIFO‐DF model

We extended the FIFO model by introducing a direct flow component, DF, representing the fraction of stroke volume that follows last‐in‐first‐out transit (Figure 1c). In this case, the expression for the RT is ${\mathrm{RT}}_{\mathrm{LV},\mathrm{DF}}={\mathrm{RT}}_{\mathrm{LV},\text{FIFO}}+\frac{\mathrm{DF}\;N_{w}\left(N_{w}-1\right)}{2}$.

#### 2.3.4 FIFO‐DF‐RV model

Finally, we added the effects of a residual volume, RV, isolated by a transport barrier from the FIFO‐DF transit pattern, which exchanges blood with the rest of the chamber by the stirring caused by random, turbulent‐like eddies. This model yields $RT_{\text{FIFO}-\mathrm{DF}-\mathrm{RV}}=\left(1-\mathrm{RV}\right)RT_{\text{FIFO}-\mathrm{DF}}+\mathrm{RV}\cdot RT_{R\;V}=RT_{\text{FIFO}-\mathrm{DF}}+\frac{2RV^{4⁄3}EDV^{1⁄3}}{3u_{l}}$. This equation represents the washout delay introduced by RV and predicts infinite RT if RV is completely isolated (Figure 1d).

### 2.4 Statistical analysis

Variables were described as mean and standard deviation unless otherwise specified. One‐way analyses of variance (followed by Dunnett's contrasts against controls) were used to compare quantitative variables among groups. Pearson linear correlation was used to assess the relationship between quantitative metrics, and root‐mean‐square‐error (RMSE) was calculated. We established statistical significance at *p* < 0.05, and all analyses were performed in R‐4.3.

## 3 RESULTS

### 3.1 Queue model predictions

**FIGURE 2:**
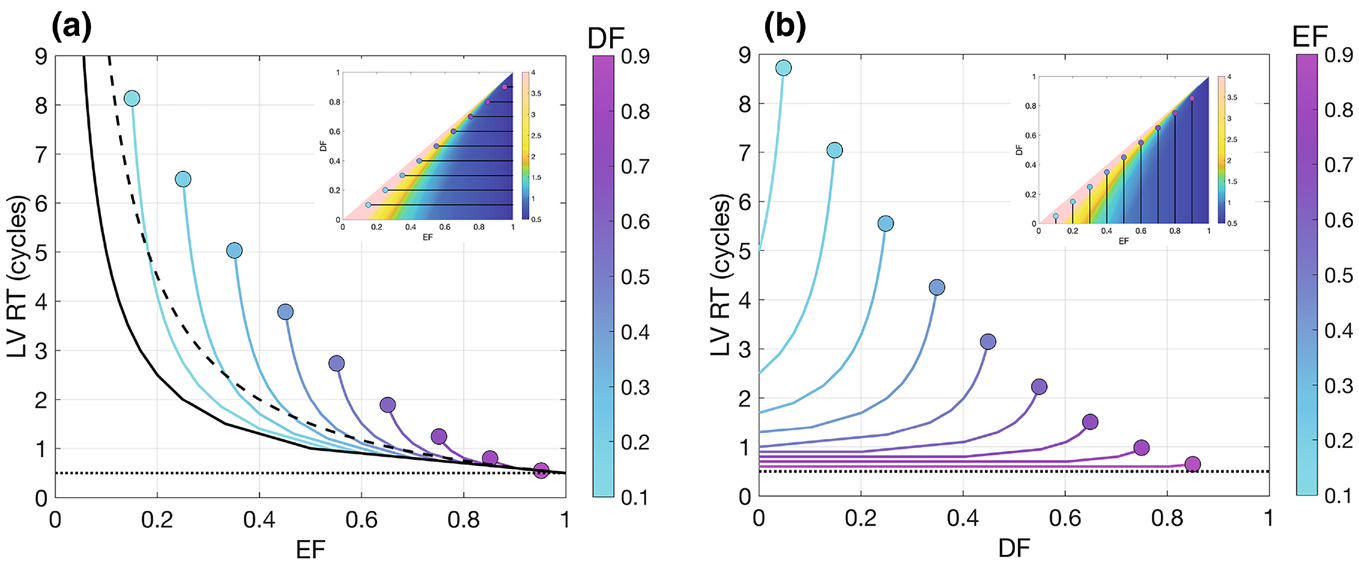
Model prediction. (a) Evolution of the RT as a function of EF for different values of DF in the FIFO‐DF model. Solid line: Zero‐mixing FIFO model. Dashed‐line: Perfect mixing model. Dotted line: $t_{0}$. (b) Evolution of RT as a function of DF for different values of EF. Dotted line: $t_{0}$.

Figure 2a shows the dependence of RT on EF for the perfect mixing (black dashed line), zero‐mixing FIFO (solid black line), and FIFO‐DF (solid color lines) models. In the FIFO‐DF case, the different curves are plotted in the range DF+0.05≤EF≤1 to allow the delayed ejection DE=EF−DF to be at least 5% of the end‐diastolic volume. In all curves, the mean RT increases as EF decreases consistent with our expectations. However, this dependency has interesting, previously unobserved features that emerge in the model. The most important feature is that, as EF approaches 1, all the curves converge to the same point $\mathrm{RT}\left(\mathrm{EF}=1\right)=t_{0}$, implying that the RT of ventricles with good systolic function is relatively insensitive to flow patterns, being almost uniquely determined by EF. In contrast, the curves diverge significantly as EF decreases, suggesting that LV flow patterns are a critical determinant of blood stasis and EF is insufficient to quantify RT in poorly contracting ventricles.

The curves in Figure 2b represent the dependence of RT on DF for constant EF. These curves indicate that, for high EF values, RT is almost independent of DF and differs little from the minimum value $t_{0}$, confirming that LVs with normal or supranormal EF are efficiently washed out regardless of flow patterns. On the other hand, the curves corresponding to low EF values grow with DF and become steeper as DF increases and EF decreases.

### 3.2 Patient characteristics

Demographic and echocardiographic data are shown in Table 1. Compared to controls, the AMI and DCM groups had lower EF (43 ± 9% and 36 ± 11% vs. 63 ± 5%, *p* < 0.001 for both), whereas it was slightly higher in the HCM group (65 ± 10%, *p* = n.s.). The AMI and DCM groups had a longer RT than controls (3.1 ± 1.0 cycles and 2.2 ± 0.8 cycles vs. 1.7 ± 0.6 cycles, *p* < 0.001) as well as larger RVs (52 ± 11% and 49 ± 15% vs. 32 ± 8%, *p* < 0.001). AMI and DCM groups exhibited lower values of DF (11 ± 7% and 5 ± 5% vs. 25 ± 8%, *p* < 0.001). In particular, DCM patients had DF values five‐fold lower than controls. HCM patients exhibited similar RT and RV to controls but had a larger DF (DF: 35 ± 14% vs. 25 ± 8%, *p* < 0.001), which was balanced by a lower RI (15 ± 9% vs. 20 ± 7%, *p* < 0.001) and DE (19 ± 8% vs. 23 ± 6%, *p* < 0.001). Among all patients, only a subset of those with DCM (40%) exhibit left bundle branch block. However, this does not lead to an increase in residence time (RT: 2.21 ± 0.94 cycles vs. 2.22 ± 0.84 cycles, *p* = 0.9).

**TABLE 1:** Clinical and imaging data.

	Overall	Control	AMI	DCM	HCM	ANOVA‐*p*
*N*	332	67	82	90	93	
Age (years old)	55 ± 16	49 ± 18	60 ± 14*	58 ± 15*	52 ± 14	<0.001
Female sex, *n* (%)	126 (38%)	35 (52%)	15 (18%)	37 (41%)	39 (42%)	<0.001
Imaging data						
Ejection fraction (%)	50 ± 16	63 ± 5	43 ± 9*	36 ± 11*	65 ± 10*	<0.001
End‐diastolic volume (mL)	105 ± 44	88 ± 25	100 ± 28	140 ± 53*	84 ± 34	<0.001
End‐systolic volume (mL)	55 ± 39	33 ± 10	58 ± 21*	92 ± 46*	30 ± 19	<0.001
Stroke volume (mL)	54 ± 19	55 ± 16	49 ± 15	55 ± 18	57 ± 25	0.2
E‐wave velocity (cm/s)	65 ± 21	70 ± 17	55 ± 17*	62 ± 19*	71 ± 25	<0.001
A‐wave velocity (cm/s)	65 ± 22	58 ± 18	69 ± 18*	71 ± 22*	62 ± 27	<0.001
LV mass (g)	196 ± 82	121 ± 30	185 ± 44*	197 ± 75*	245 ± 97*	<0.001
Stasis and transport data						
LV blood residence time (cycles)	2.3 ± 1.0	1.7 ± 0.6	3.1 ± 1.0*	2.2 ± 0.8*	1.9 ± 1.3	<0.001
Direct flow (%)	19 ± 15	25 ± 8	11 ± 7*	5 ± 5*	35 ± 14*	<0.001
Retained inflow (%)	19 ± 8	20 ± 7	17 ± 6	24 ± 8*	15 ± 9*	<0.001
Delayed ejection (%)	21 ± 7	23 ± 6	19 ± 6*	22 ± 7	19 ± 8*	<0.001
Residual flow (%)	42 ± 15	32 ± 8	52 ± 11*	49 ± 15*	31 ± 13	<0.001
Model's performance						
Perfect mixing, *R* (RMSE)	0.37 (1.07)	0.10 (0.72)	0.28 (1.36)	0.63 (1.11)	0.10 (0.77)	
FIFO, *R* (RMSE)	0.20 (1.72)	0.12 (0.97)	0.10 (2.22)	0.61 (0.68)	0.18 (2.33)	
FIFO‐DF, *R* (RMSE)	0.37 (1.15)	0.09 (0.84)	0.26 (1.72)	0.64 (0.62)	0.09 (0.86)	
FIFO‐DF‐RV, *R* (RMSE)	0.62 (0.88)	0.70 (0.43)	0.60 (0.78)	0.80 (1.31)	0.40 (0.82)	

Abbreviations: AMI, acute myocardial infarction; DCM, dilated cardiomyopathy; HCM, hypertrophic cardiomyopathy; LV, left ventricle; R, correlation coefficient; RMSE, root mean squared.

* *p* < 0.05 versus Control group in Dunnett's contrasts.

**FIGURE 3:**
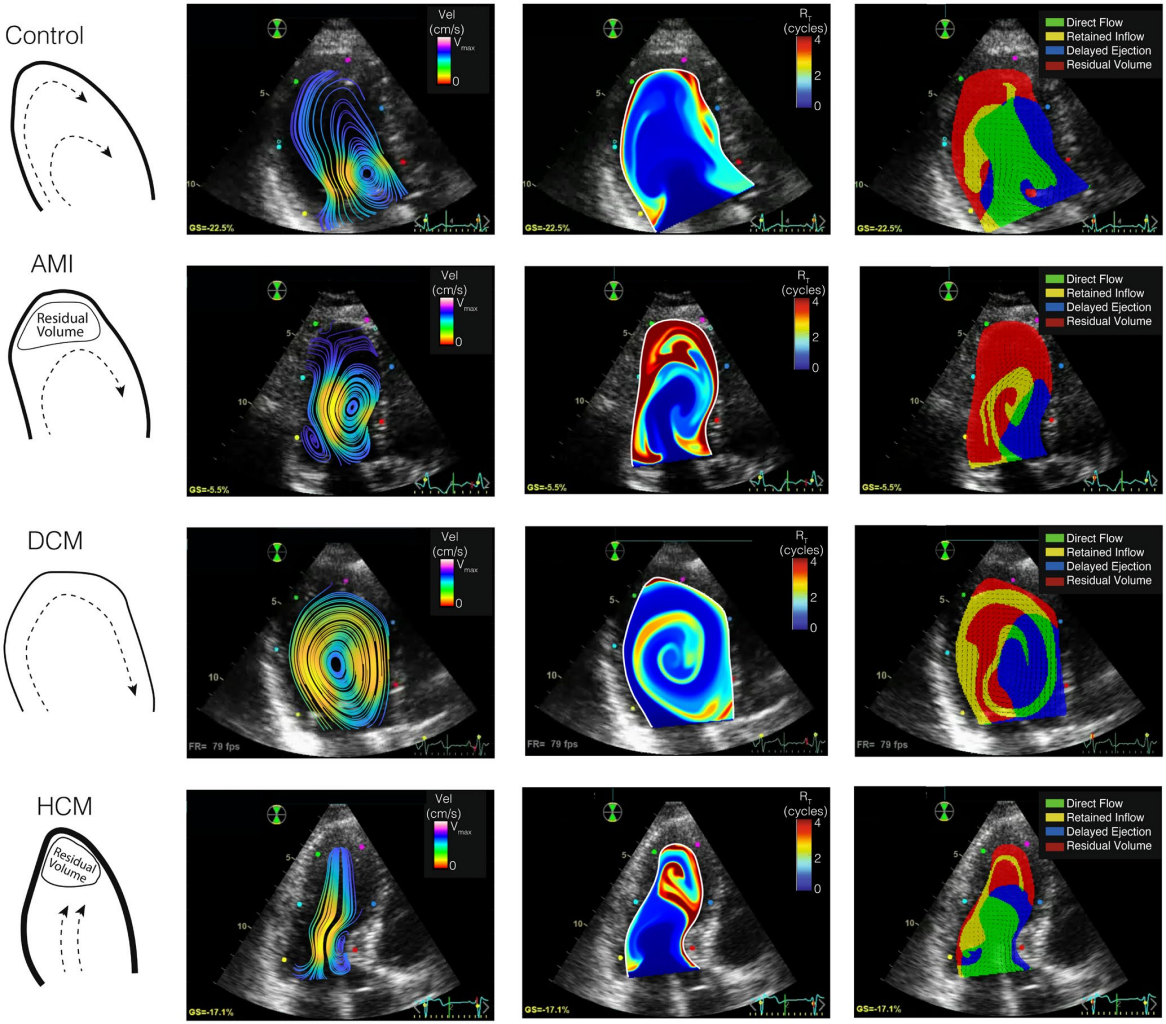
Examples of flow, RT and Transport. Sketch of main flow patterns (1st column) and examples of the 2D + t flow fields (2nd column), RT maps (3rd column) and blood transport barriers (4th column) for the control group (1st row), AMI patients (2nd row), DCM patients (3rd row), and HCM patients (4th row).

Figure 3 shows instantaneous flow streamlines, RT maps, and flow components from representative individuals of the four groups. DCM patients had larger and stronger swirling patterns than controls, while these patterns were weaker in HCM patients.

### 3.3 Clinical validation of queue models

**FIGURE 4:**
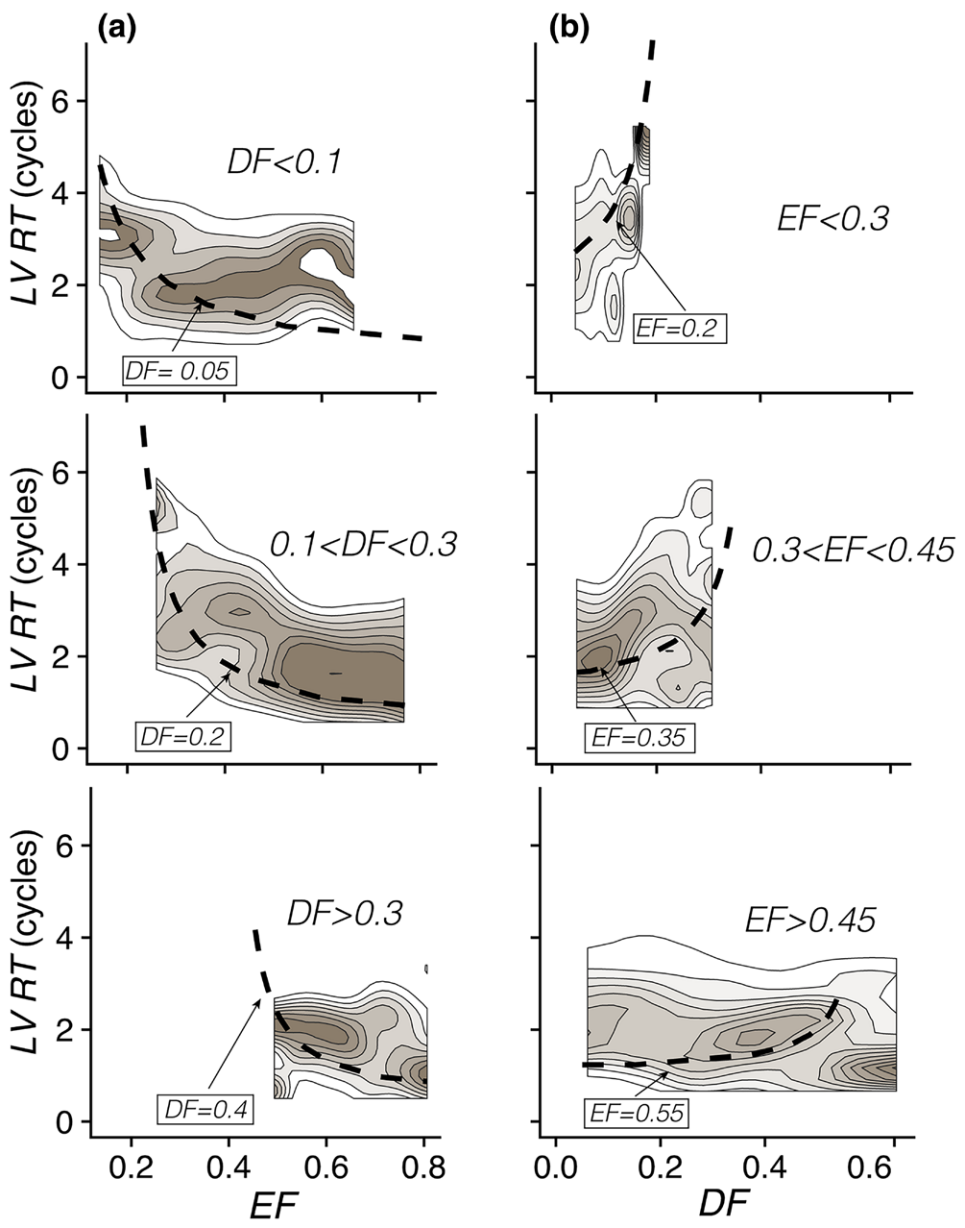
Residence time in the studied subjects. (a) Conditional probability density plot of RT in all the subjects as a function of EF. Data is split for low (<0.1), moderate ([0.1, 0.3]), and large (>0.3) DF. (b) Conditional probability density plot of RT in all the subjects as a function of DF. Data is split for low (<0.3), moderate ([0.3, 0.45]), and high (>0.45) EF. In both panels, the dashed line represents the FIFO‐DF model for the median of the data shown.

We tested whether the key predictions of LV transit queue models reported above are reproduced in the clinical setting. Figure 4a shows the probability distributions of RT conditional to EF from all study subjects aggregated in three groups depending on the patient‐specific value of DF: low DF (DF < 0.1, top panel), intermediate DF (0.1 < DF <0.3, middle panel), and high DF (DF > 0.3, bottom panel). The dependence of these distributions on EF was reasonably well captured by the FIFO‐DF queue model when DF was set to each group's median value (i.e., 0.05, 0.2, and 0.4 for the low‐, intermediate‐, and high‐DF groups). Patient data confirmed that RT increases as EF decreases and that this decrease was less pronounced in patients with a lower DF component. To focus on the effect of DF on RT, we plotted the probability distributions of RT conditional to DF for our study population (Figure 4b). In this case, the data was also aggregated in three groups, depending on the patient‐specific value of ejection fraction: low EF (EF < 0.1, top panel), intermediate EF (0.3 < DF <0.45, middle panel), and normal EF (EF >0.45, bottom panel). As in Figure 4a, the FIFO‐DF model predictions for each group's median value of EF were included for reference. Once again, the patient distribution followed well the FIFO‐DF model predictions. In particular, the steep increase of RT with DF for low EF was distinctly observed in the clinical data. Additionally, a clear decline in the sensitivity of RT to DF was adequately identified.

**FIGURE 5:**
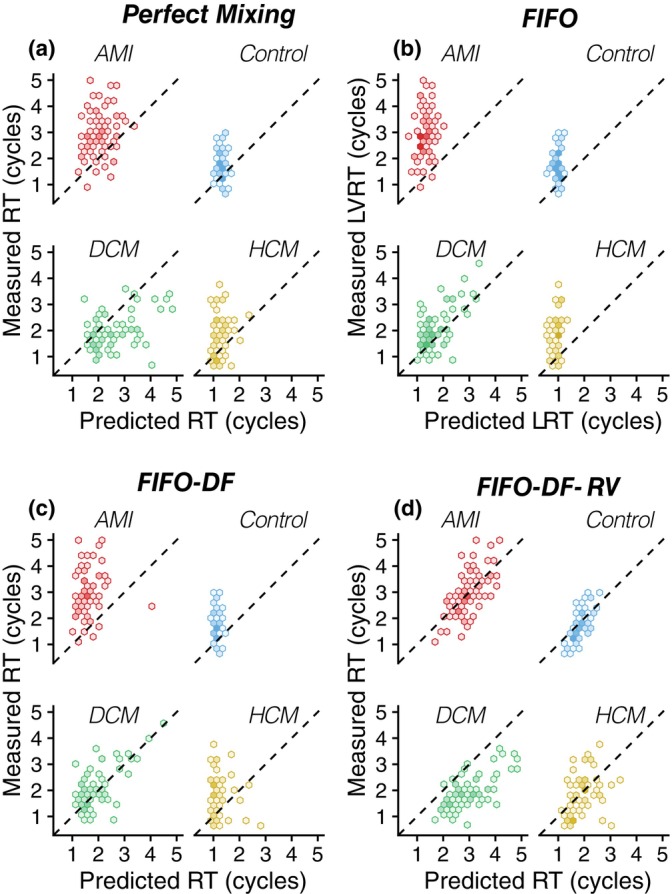
Hexagonal heatmap plot, shaded by data density, of the prediction and measured RT for the perfect mixing (a), zero‐mixing FIFO (b), FIFO‐DF (c) and the FIFO‐DF‐RV (d) models, respectively. Data are colored by group. AMI, acute myocardial infarction; DCM, dilated cardiomyopathy; HCM, hypertrophic cardiomyopathy.

Figure 5a displays the measured RT values versus the RT values predicted with the perfect mixing model. This model offered a compromise fit for the entire population, yielding a modest correlation coefficient of *R* = 0.37 and a *RMSE =* 1.07 cycles. FIFO and FIFO‐DF model predictions correlated better with measured RT values, especially in the DCM group (*R* = 0.61 and 0.64, RMSE = 0.68 and 0.62 cycles, respectively), although they underestimated the RT of many patients in the control, HCM, and AMI groups (see Figure 5b, c and Table 1). Finally, the FIFO‐DF‐RV model (Figure 5d) predicted the RT of the AMI and HCM groups better than the other models (*R =* 0.60 and 0.40, RMSE = 0.78 and 0.82 cycles). However, it overestimated the RT of the DCM group (mean difference = −1.14 cycles).

## 4 DISCUSSION

This work presents queue models of LV transit to examine the relationship between RT, LV ejection fraction, and direct flow (DF) and residual volume (RV) components (Eriksson et al., 2010). We found this relationship less intuitive than previously thought for several reasons. First, flow transport efficiency affects RT differently depending on global systolic chamber function. A ventricle with an EF near 100% cannot have extensive stasis regardless of intraventricular flow patterns because most of its volume is cleared each cardiac cycle. And in LVs with reduced EF, stasis can still be moderate if the LV flow establishes a continuous path for all incoming blood to reach the LV outflow tract within a few cardiac cycles. Conversely, stasis can be significant for normal EF values if a transport barrier excludes significant regions from the main transit path. Along these lines, the definition of RV as blood with RT > 2 cycles may cause ambiguity between blood in slow transit and truly stagnant blood (Hendabadi et al., 2013). Besides, it is difficult to interpret DF without considering systolic function since EF=DF+DE≥DF. This interdependency may obscure the comparison of flow components between healthy and diseased groups. The low values of DF reported in impaired ventricles (Bermejo et al., 2015; Demirkiran et al., 2022; Eriksson et al., 2010, 2013; Postigo et al., 2022; Stoll et al., 2019) could merely mirror their lower EF instead of indicating changes in flow efficiency. Likewise, a concurrent increase of DF and EF is observed following dobutamine infusion (Sundin et al., 2020).

Consistent with the ideas discussed above, our queue models show that RT does not only increase as EF decreases. It also becomes sensitive to additional factors reflecting the overall LV transit pattern and flow‐induced mixing. Notably, these models suggest the mean LV RT increases with DF when EF is held constant. While perhaps not intuitive, this finding reflects that increasing DF promotes more incoming blood to exit the chamber immediately while retaining the resident blood pool for more cycles. The trend is also observed in clinical data when examining RT versus DF for constant EF values.

Queue models offer mathematical expressions for RT that can be particularized to patient‐specific EF, DF, and RV values. Although the models' simplifications and parameterization limit their predictive accuracy, comparing their predictions with measurements still provides valuable insights. Such comparisons not only help to assess the models' reliability but also provide a deeper understanding of the underlying physiological phenomena. For instance, we found that including a transport barrier was essential to reproduce patient‐specific RT values in the AMI group. In line with this finding, significant stagnant regions are often found near the LV dyskinetic apex in AMI patients (Corrado et al., 2019). In contrast, low‐DF FIFO models performed best in the DCM group, consistent with a dominant role of the large swirling pattern typically found in enlarged ventricles. In dilated ventricles with low EF, this large‐scale swirl allows the entire blood pool to slowly transit from inflow to outflow, even if some of this blood is labeled as RV using the definition RT >2 cycles. Previous works highlighted the relevance of this flow structure, proposing it impairs mechanical energy efficiency (Abe et al., 2013; Gharib et al., 2006) in connection with the reduction of the DF component (Mangual et al., 2013). On the other hand, since the FIFO transit pattern achieves minimum RT at any given EF, our models and data suggest that, under particular geometries, ventricular dilation may favor a blood flow pattern protective against mural thrombosis. This idea is supported by ventricles with DCM having shorter RTs than those with AMI when matched by EF. Whether this phenomenon reflects functional or evolutionary adaptations that favor blood clearance over mechanical energy conservation is debatable. However, it underscores that not all forms of remodeling in cardiac disease are equally detrimental. This could be particularly relevant in patients with heart failure and preserved ejection fraction (HFpEF). Chambers with this condition are typically small due to concentric remodeling, hampering diastolic vortex formation. In addition, as in HCM, advanced diastolic dysfunction in HFpEF increases late filling and, consequently, DF. Although not tested in the present study, it is therefore likely that the FIFO‐DF‐RV would also perform best in this condition. These results highlight that , despite their seemingly simple foundations, queue models effectively capture the diverse flow patterns that lead to blood stagnation in left ventricles of various shapes and sizes.

### 4.1 Clinical implications

The models and data herein described explain why EF alone is a poor biomarker for primary prevention of LV mural thrombosis and cardioembolism due to LV blood stasis. Thus, suboptimal patient selection may have hampered a favorable risk–benefit ratio of landmark clinical trials of stroke prevention (Ntaios et al., 2019). Dependency on functional barriers that modulate intraventricular flow transit is a key determinant of the risk of mural thrombosis in patients with abnormal LVs. LV regional wall motion, atrioventricular electrical coupling, and LV inflow geometry are well‐known determinants of intraventricular flow transit. Consequently, our results explain why therapeutic procedures such as cardiac resynchronization therapy (Rossini et al., 2017), mitral edge‐to‐edge repair (Tichelbacker et al., 2022), and valve replacement (Vu et al., 2019) may induce intraventricular stasis despite a favorable effect on EF. For the reasons discussed above, this will not be an issue in patients with normal EF, even in the presence of overt regional wall motion abnormalities (Rodriguez‐Gonzalez et al., 2025). However, in patients with a low EF, flow imaging modalities are necessary to measure flow transit and/or stasis and eventually account for the risk of intracardiac thrombosis.

### 4.2 Limitations

The models include two free parameters: $t_{0}$, representing diastole duration normalized by the cardiac period, and α, accounting for the fraction of blood exchanged in the RV during each cardiac cycle. Both parameters are set constant for simplicity. However, to address the influence of α on RT, we derived a phenomenological extension dependent on end‐diastolic volume, RV, and a characteristic velocity, $\;u_{l}$, also fixed based on literature.

The limitations of the VFM modality, used to evaluate our models with clinical data, have been discussed thoroughly in previous publications (Asami et al., 2017; Avesani et al., 2021; Garcia et al., 2010; Hvid et al., 2023). In short, VFM assumes planar flow in the long‐axis apical view of the LV, an approximation that has been justified by several groups (Asami et al., 2017; Avesani et al., 2021; Garcia et al., 2010; Hvid et al., 2023). Also, flow compartments and residence time have been reported to be well captured using VFM when compared to the reference 4D flow phase contrast magnetic resonance data (Postigo et al., 2022).

Finally, in the queue models, RT was calculated assuming mixing and transport processes span during infinite number of cycles $N_{\text{cycles}}\to \infty$. However, the number of cycles used to compute RT from VFM data is necessarily finite. We selected $N_{\text{cycles}}=8$ based on observations that RT remains unchanged in normal LVs after 8 cycles (Tichelbacker et al., 2022). We ensured that this discrepancy did not cause significant differences by also evaluating our queue models for $N_{\text{cycles}}=8$ (Figure S1). Except for unrealistically low EF values, the models produced similar results for $N_{\text{cycles}}=8$ and $N_{\text{cycles}}\to \infty$.

## 5 CONCLUSIONS

Simple queue models of flow transit illustrate how intraventricular stasis becomes increasingly sensitive to flow patterns as EF decreases. Since thrombosis is most likely with blood stasis, this result may explain some of the limitations of EF to predict LV thrombus and stroke in patients with impaired systolic function. Our models suggest that LV transit efficiency is paradoxically impaired when more incoming blood exits the chamber immediately, thus retaining the pool of resident blood longer. Particular flow patterns—like swirling flows in dilated spherical ventricles—are related to lower values of RT and, consequently, may lower cardioembolic risk.

## AUTHOR CONTRIBUTIONS

P.M.‐L., J.C.A., and J.B. conceived and designed research; analyzed data; interpreted results of experiments; prepared figures; drafted manuscript; edited and revised the manuscript; and approved final version.

## FUNDING INFORMATION

This work was supported by the National Institutes of Health under grants 1R01HL160024 and 1R01HL158667, the Spanish Research Agency and the Fulbright Program (PRX22/00606‐PS00365432), the Instituto de Salud Carlos III and the European Regional Development Fund under the grant PI21/00274‐PACER1.

## CONFLICT OF INTEREST STATEMENT

P.M.‐L., J.C.A., and J.B. are inventors of a method for quantifying intracardiac stasis and shear stresses from imaging data under a Patent Cooperation Treaty application (WO2017091746A1).

## 6 ETHICS STATEMENT

The Institutional Ethics Committee of the Hospital Gregorio Marañón (Madrid, Spain) approved these studies, and all participants provided written informed consent.

## Supporting information


Appendix S1.

## Data Availability

The data that support the findings of this study are available from the corresponding author upon reasonable request.
