# Supplementary material for: Flow transport and not ejection fraction determines blood stasis in patients with impaired left ventricular systolic function
Source: Physiol Rep. 2025 Jul 4;13(13):e70351. doi: 10.14814/phy2.70351 (PMC12227659; doi:10.14814/phy2.70351)
Supplement: Supplementary file 1 — Appendix S1. [file PHY2-13-e70351-s001.pdf]

The role of flow transport and chamber function on left ventricular stasis

Pablo Martinez-Legazpi[1],
Javier Bermejo[2],
Juan C. del Alamo[3]

[1]Department of Mathematical Physics and Fluids, Facultad de Ciencias, Universidad Nacional de Educación a Distancia, UNED and CIBERCV, Madrid, Spain.
[2]Department of Cardiology, Hospital General Universitario Gregorio Marañón; Facultad de Medicina, Universidad Complutense de Madrid, Instituto de Investigación Sanitaria Gregorio Marañón and CIBERCV, Madrid, Spain.
[3]Mechanical Engineering Department, Division of Cardiology, and Center for Cardiovascular Biology, University of Washington, Seattle, WA, USA.

ADDITIONAL ONLINE MATERIAL

Additional Online Methods

1. Queue Models for LV residence time

This section presents several models to estimate the mean RT in the LV, accounting for different LV transit patterns and microscopic mixing, using EF as the independent variable. The models are presented in order of increasing complexity, starting with the simplest mixing hypothesis and progressively incorporating flow information by adding the contributions of DF, given that $DF + DE = EF$, and RV to EF. Although some of these models obviously oversimplify blood transit in the LV, we believe that their sequential derivation helps to understand better how blood transits the LV. By comparing how different models fit different cohorts, we aim to shed light onto the dominant LV transit pattern in these groups.

1.1. Perfect mixing transit model. The mean LV RT can be calculated as a function of the EF alone by assuming that blood inside the chamber achieves perfect microscopic mixing at the end of diastole. While this assumption does not accurately reflect flow transit inside the human LV (12), it allows to make simple model predictions that can serve as reference for the rest of the models developed in this study. It also provides the simplest introduction to the more complex transit models derived in this section. Following the diagram in **Figure 1A**, the mean LV residence time under perfect mixing can be obtained in closed form starting with the recursive formula

$$RT_{LV,n+1} = (1 - EF)(RT_{LV,n} + 1) + EF \cdot t_0 \quad [e2]$$

where the parameter $t_0 < 1\ cycle$ represents the duration of diastole (more precisely, it is the mean RT of the blood that enters the LV each cycle at the end of diastole). This parameter also appears in all the other models and, while it may vary slightly from patient to patient, we fixed it to a constant value $t_0 = 0.7$ cycles for the sake of simplicity. Following *e2* to the limit $n \to \infty$, and noting that $(1 - EF)^n \to 0$ in that limit, we obtain

$$RT_{LV,mixed} = \sum_{n=0}^{\infty} (1 - EF)^n [1 - EF(1 - t_0)] = t_0 + \frac{1 - EF}{EF} \quad [e3]$$

after some manipulation. When the EF approaches one, the model yields $RT_{LV} \approx t_0$. On the other hand, the model predicts that RT_{LV} becomes infinitely high in the limit when EF tends to zero.

1.2. Zero mixing with perfect FIFO transit model (FIFO). For any given EF value, the mean RT in the LV is minimized when blood transits the chamber following a first-in first-out (FIFO) flow

pattern where blood with the highest RT immediately exits the chamber. Qualitatively, the FIFO pattern is justified by the existence of diastolic vortices that redirect the inflow to outflow. The opposite transit pattern, i.e., last-in first out or LIFO, leads to infinite RT since it flushes incoming blood while indefinitely retaining blood already present in the chamber. For this reason, the zero-mixing LIFO model is trivial, and we will only consider the FIFO model here. Assuming zero mixing, the zero-mixing FIFO model partitions the LV chamber into $N_w = int\left(\frac{1}{EF}\right)$ equal queued compartments of volume $V_{i,EF} = EF \cdot EDV$, as shown in **Figure 1B**. We denote N_w the washout number as it measures the number of cardiac cycles required to clear the LV completely. RT inside each of the queued N_w compartments is $RT_{i,EF} = t_0 + i - 1$ cycles. Since the EDV is usually not an exact multiple of $V_{i,EF}$, there will be a smaller, residual ejection compartment with volume $V_{\Delta\mathrm{E}} = EDV \cdot (1 - EF \cdot N_w)$ and residence time $RT_\Delta = t_0 + N_w$ (**Figure 1B**). The mean residence in the LV using this model, can be written as:

$$RT_{LV,FIFO} = EDV^{-1}\left(V_{\Delta\mathrm{E}} \cdot RT_{i,\Delta} + \sum_{i=1}^{N_w} V_{i,EF} \cdot RT_{i,EF}\right) = N_w + t_0 - EF(N_w + 1)N_w/2. \qquad [e4]$$

In *e4*, the only independent variable is EF and the parameter t_0 is fixed, as described above. When $EF = 1$, N_w is also equal to one and we obtain $RT_{LV} \approx t_0$, like in the perfect mixing model. Below, we will see that the zero-mixing FIFO model is a particular case of a more general model that involves EF and DF when the direct flow component is fixed to be zero, i.e., $DF = 0$. On the other hand, the LIFO model is obtained when $DF = EF$.

1.3. Imperfect FIFO transit with direct flow component (FIFO-DF): We next consider a zero-mixing model where a fraction of the stroke volume is a direct flow compartment exhibiting LIFO transit. This imperfect FIFO model is characterized by two independent variables, i.e., EF and DF. Since $EF = DF + DE$, where the delayed ejection $DE \geq 0$, the model is constrained to the $DF \leq EF$ half plane in the space of independent variables. As before, t_0 is considered constant. Following the same reasoning, we used in the ideal FIFO case one may obtain a closed-form relationship for the mean RT. However, in this case the washout number is $N_w = int\left(\frac{1-DF}{EF-DF}\right)$, the equal queued compartments have volumes $V_{i,EF,DF} = (EF - DF) \cdot EDV$, and the residual ejection compartment has a volume $V_{\Delta\mathrm{E}} = EDV \cdot (1 - DF - (EF - DF) \cdot N_w)$ (see **Figure 1C**). In this case, the LV mean residence time is given by

$$RT_{LV,DF} = EDV^{-1}\left(DF \cdot t_0 + V_{\Delta\mathrm{E}} \cdot RT_{i,\Delta} + \sum_{i=1}^{N_w} V_{i,EF} \cdot RT_{i,EF}\right) = \qquad [e5]$$

$$= (1 - DF) \cdot N_w + t_0 - (EF - DF) \cdot (N_w + 1)N_w/2 = RT_{LV,FIFO} + DF\, N_w(N_w - 1)/2$$

The rightmost side of this expression shows LV washout is delayed by the direct flow component and that the delay is roughly proportional to DF and inversely proportional to the square of the ejection fraction. It is also straightforward to see that $RT_{LV,DF}$ becomes infinitely high when $DF = EF$ because $N_w \to \infty$. Also interesting, the model recovers that $N_w = 1$ when the ejection fraction becomes one, so that $RT_{LV,DF}(EF = 1) = t_0$ regardless of the direct flow component, converging with the other two models derived above.

1.4. FIFO-DF with a transport barrier and residual volume (FIFO-DF-RV): We expanded the FIFO-DF model described above to quantify how residual volumes isolated by transport barriers affect RT. Following the diagram of **Figure 1D**, the mean LV residence time in this case is given by

$$RT_{FIFO-DF-RV} = (1 - RV)RT_{FIFO-DF} + RV \cdot RT_{R\,V},. \qquad [e6]$$

where RV is the residual volume component and RT_{RV} is the mean residence time inside the residual volume. Several working expressions can be obtained for RT_{RV}, allowing for better interpreting this model and reducing its dependence on additional parameters. First, assuming that blood inside the RV has residence time high enough to be well mixed, the RT in the residual volume can be expressed as

$$RT_{R\,V} = RT_{FIFO-DF} + \frac{1-\alpha}{\alpha} = RT_{FIFO-DF} + \Delta RT, \qquad [e7]$$

where α is the fraction of blood within the residual volume that is exchanged with the rest of the blood pool inside the LV. We note that this exchange's contribution to $RT_{FIFO-DF}$ was neglected for simplicity, since it only introduces a small, second-order effect. Another interpretation of the model is made possible by defining $\Delta RT = \frac{1-\alpha}{\alpha}$ as the increment in RT inside the residual volume with respect to its value in the rest of the LV chamber. Combining the expressions above, we have:

$$RT_{FIFO-DF-RV} = RT_{FIFO-DF} + RV \cdot \frac{1-\alpha}{\alpha} = RT_{FIFO-DF} + RV \cdot \Delta RT, \qquad [e8]$$

indicating that $RT_{FIFO-DF-RV} = RT_{FIFO-DF}$ when there is no residual volume ($RV = 0$) or if RV is fully exchanged with the rest of the chamber every cardiac cycle ($\alpha = 1$). On the other hand, $RT_{FIFO-DF-RV} \to \infty$ when the residual volume is fully isolated and $\alpha \to 0$ (or equivalently, $\Delta RT \to \infty$). It is important to note that RV can be non-zero in the absence of transport barriers. For instance, a FIFO model with $EF < 0.5$ has a region with $RT_\Delta > 2$ cycles, which constitutes a

residual volume according to its original definition. However, this residual volume is in continuous transit rather than isolated beyond a barrier.

While the previous expression is useful to understand the model, it depends critically on an additional parameter (α) that is difficult to quantify. In an effort to minimize and clarify this dependence, we will estimate $RT_{R\,V}$ under the assumption that it is determined by diffusion with an effective diffusion coefficient $\kappa = u_l$, where u_l is the characteristic velocity of small-scale eddies in the LV and l is an effective mixing length (35, 36). Reasonable estimates for these quantities are $u_l \approx 0.1\ m/s$ and $l \approx (EDV \cdot RV)^{1/3}$ (37). We then take the simplified, 1D version of the governing equation for residence time with diffusion, under no flow conditions, and in steady state equilibrium (19),

$$0 = \kappa \frac{\partial^2 T_R}{\partial x^2} + 1. \qquad [e9]$$

Equation *e9* can be solved imposing as boundary conditions $\frac{\partial^2 T_R}{\partial x^2}(0) = 0$, i.e., zero diffusion of residence time and the LV wall, and $T_R(l) = RT_{FIFO-DF}$, i.e., that residence time at the interface of the residual volume matches the mean value in the rest of the chamber. The solution is $T_R(x) = RT_{FIFO-DF} - (x^2 - l^2)/(2\kappa)$. The spatially averaged value inside the residual volume is estimated as

$$RT_{RV} = \frac{1}{l}\int_0^l T_R(x)dx = RT_{FIFO-DF} + \frac{2l^2}{3\kappa} = RT_{FIFO-DF} + \frac{2l}{3u_l}, \qquad [e10]$$

so that

$$RT_{FIFO-DF-RV} = RT_{FIFO-DF} + \frac{2RV^{4/3}EDV^{1/3}}{3u_l}, \qquad [e11]$$

which is independent of the value of α. Similar to equation *e5*, this expression provides the LV washout delay associated to the residual volume.

Additional Online Figure

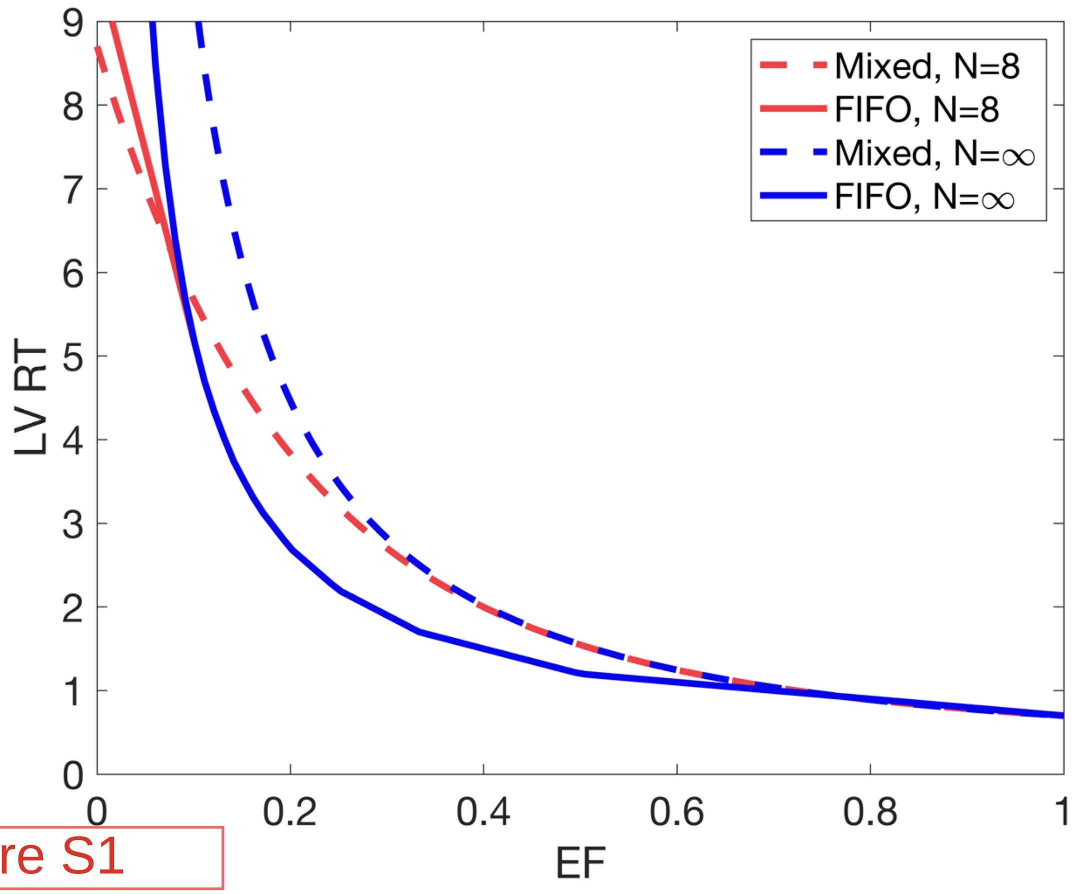

Figure S1

AO Figure 1: Influence of the number of studied cycles in the calculation of LVRT using the perfect mixing model (dashed line) and the FIFO model (solid line) for N= 8 cycles (in red) and in the limit of infinity (in blue).
